# Use of Human Habitat in an Extreme Desert by the Generalist Red Fox: Risk and Energy Considerations

**DOI:** 10.1002/ece3.74392

**Published:** 2026-09-16

**Authors:** Ron Efrat, Nitzan Segev, Oded Berger‐Tal

**Affiliations:** ^1^ Department of Migration Max Planck Institute of Animal Behavior Radolfzell Germany; ^2^ Science Division Israel Nature and Parks Authority Jerusalem Israel; ^3^ Mitrani Department of Desert Ecology, Jacob Blaustein Institutes for Desert Research Ben‐Gurion University of the Negev Midreshet Ben‐Gurion Israel

**Keywords:** arid habitats, conservation behavior, energy landscape, human–wildlife interactions, landscape of fear, movement ecology

## Abstract

Human‐induced environmental changes are widespread, affecting the natural world at varying scales, from individual animals to entire ecosystems. In human‐modified habitats, animals are expected to change their behavior in ways that depend on both environmental and species‐specific characteristics. We studied the effects of human‐modified habitats on the behavior of the generalist Red Fox, *Vulpes vulpes* , in a hyper‐arid desert, where the contrast between natural and human‐modified habitats is strong. We used GPS tracking to investigate the movement of 10 foxes in relation to human habitat between December 2022 and December 2024. We examined how environmental factors affected the selection for human habitats by foxes, the potential costs of visiting these habitats and whether these costs shaped the rate of visitation. We found that most foxes maintained territories in natural areas and only visited human habitats at night. Overall visitation rate increased with reduced relative humidity, while other environmental variables had only marginal effects. Individual visitation rates decreased as the distance of a fox territory from human habitat increased, ranging from almost nightly visits to a visit every three nights. At the same time, up to a threefold increase in movement was detected when comparing nights with and without visits to human habitats depending on the territory distance from these habitats. Our results suggest that the rate of human habitat use by the Red Fox in desert environments is shaped by a trade‐off between elevated risk and energy expenditure related to longer movements and the benefits of using these habitats. Understanding the determinants of this trade‐off in extreme environments improves our understanding of animal behavioral ecology and can support the design of human–wildlife conflict mitigation strategies.

## Graphical abstract

In the hyper‐arid southern Jordan Rift Valley, Red Foxes predominantly maintain territories in natural habitats but frequently visit human‐modified areas at night to access resources. The study reveals a significant behavioral trade‐off where foxes balance the benefits of human‐provided resources against the elevated energy expenditure and potential risks associated with traveling greater distances.

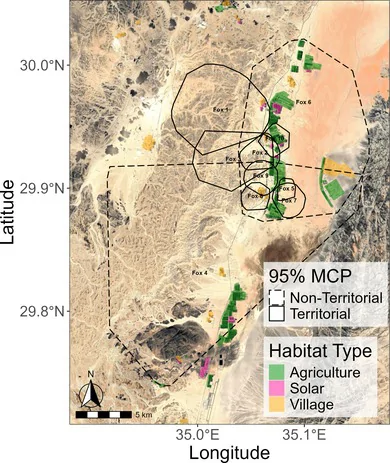

## 1 Introduction

Animal movement is a key behavior that affects all aspects of life, including the acquisition of resources by the animal, its energy balance, and its risk management (Nathan et al. 2008). Human‐induced changes to the natural environment modify the costs and benefits associated with animal movements, resulting in variations in the movement patterns of animals (Efrat et al. 2025; Fahrig 2007; Oro et al. 2013; Teitelbaum et al. 2026). Human habitats often increase the availability of resources in a landscape (Oro et al. 2013). This can induce long movements, when resource‐rich patches create landscape (resource) supplementation that supports populations of species traveling to and from these patches (Dunning et al. 1992). Contrastingly, a study covering 57 mammal species has shown that movement ranges of individuals decrease with a higher human footprint, suggesting that an increase in resource availability overall leads to a decrease in animal movement (Tucker et al. 2018). The large variation shown in the responses of animals to human‐modified habitats may be explained by species‐specific characteristics, the types of human‐induced changes to the environment, and the natural environment in which they occur (Cooke et al. 2019; Dharmarajan et al. 2021; Sih et al. 2011).

Arid environments, characterized by low productivity, present an extreme example of the contrast between human‐modified and surrounding natural environments (Oswald et al. 2025). Species living in desert and arid environments display physiological, morphological and behavioral adaptations to these extreme habitats (Willmer et al. 2009). Such adaptations can be divided into fixed and flexible, roughly dividing species into specialists and generalists (Berger‐Tal and Saltz 2016; Rymer et al. 2016). For example, some species survive in these harsh environments by specializing in taking advantage of locally abundant resources, while other species survive using a large variety of less abundant resources (Lehmann et al. 2013). Fixed adaptations can be efficient in a stable environment, but in the case of rapid human changes they may prevent species from taking advantage of the new resources (Diamant et al. 2025; Sih et al. 2011). Furthermore, human activity in arid environments can produce local resource hotspots that have far reaching consequences for the local species and their behavior, depending on how flexible these species are (Diamant et al. 2025).

While the evolutionary history and specialization of a species can predict its response to human‐induced changes to the environment (Sih et al. 2011), local trade‐offs are expected to impact the behavior of individuals. The use of human habitats can expose individuals to more resources, but also to boosted inter‐ and intra‐species competition, novel risks, and increased energy expenditure stemming from a rise in required movement or the costs of such movements (Newbold et al. 2015; Oro et al. 2013; Shepard et al. 2013). Desert environments potentially increase the benefits of using human habitats due to the contrast in resource availability between the human‐modified and natural habitats. However, desert environments also increase the risks of using human habitats due to high movement costs associated with heightened temperatures, low humidity, and the scarcity of food and water. To examine this possible trade‐off, we studied the movement patterns of the generalist Red Fox, *Vulpes vulpes* , in one of the most extreme climates in which the species is found, the hyper‐arid southern Jordan Rift Valley.

The Red Fox is a medium‐sized carnivore naturally found throughout most of the Northern Hemisphere (Macdonald and Sillero‐Zubiri 2004). The diet of the Red Fox is diverse and adaptive, with the species known to frequent human‐altered habitats and even live in large cities (Bateman and Fleming 2012; Castañeda et al. 2022). The southern Jordan Rift Valley is a hyper‐arid desert, where temperatures typically rise to over 40°C in summer and annual rainfall rarely exceeds 30 mm. To survive these extreme conditions, foxes adapt their behavior and diet by, for example, feeding on water‐rich fruit and vegetables (Geffen and Girard 2003). However, being highly adaptive, the Red Fox is a natural candidate to ease such adaptations by using available resources in human habitats in such environments. Yet how the interaction of foxes with human habitat in desert environments is shaped and how it affects their behavior is still unknown.

To better understand this interaction, we investigated the movement of foxes in and around human habitats in the southern Jordan Rift Valley using GPS collars, tracking individual foxes for up to 2 years and analyzing their movement patterns. We asked the following questions: (1) What are the rates of visitation of foxes to human‐altered areas in a hyper‐arid desert environment? (2) What natural environmental factors affect these rates? (3) What are the movement‐related costs of these visits, and do they affect the rates of visitation? Although we could not directly measure costs and benefits, we use well established proxies and aim to shed light on how human‐induced environmental changes affect the ecology and behavior of species in challenging desert environments.

## 2 Materials and Methods

### 2.1 Study Area

**FIGURE 1:**
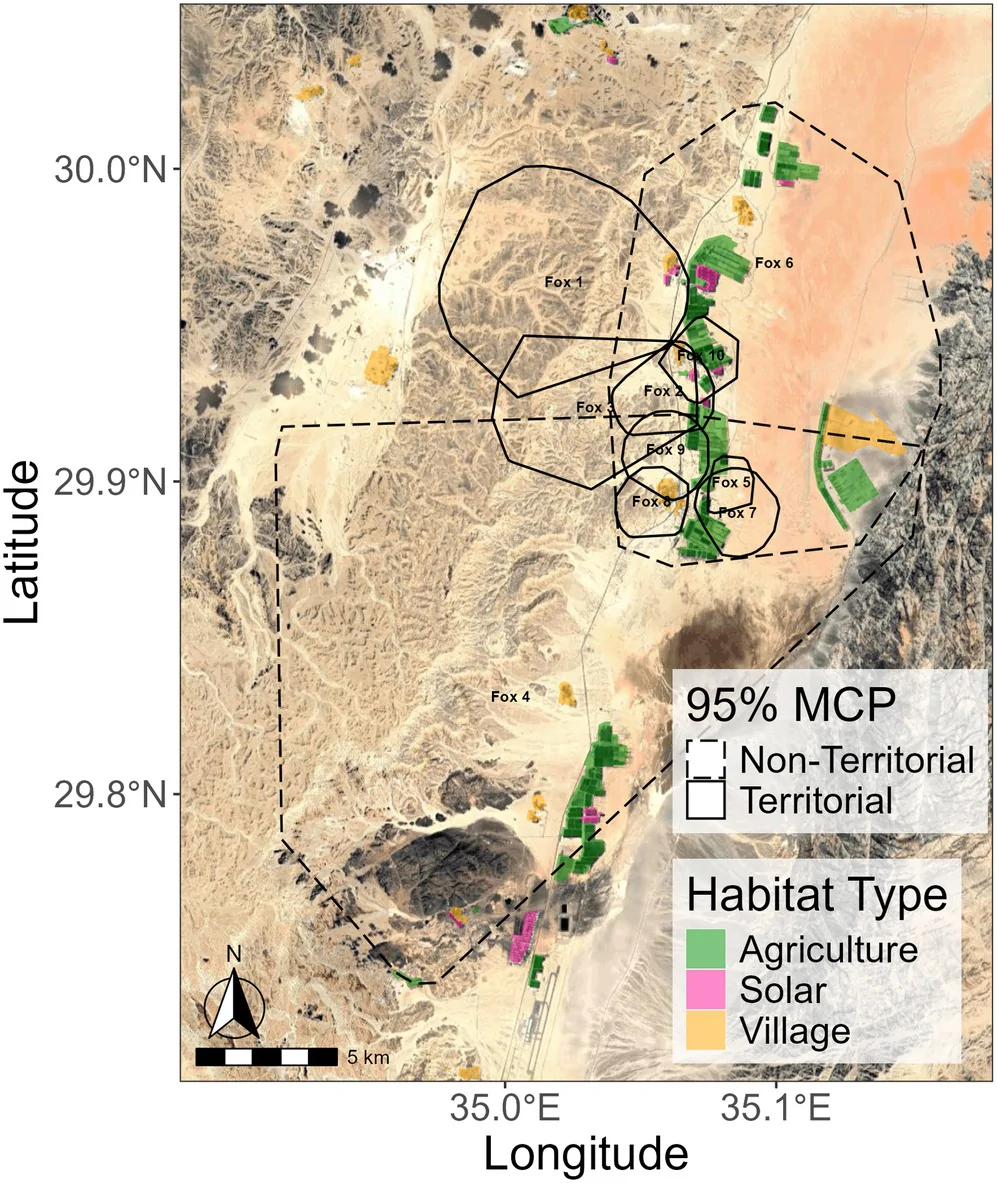
Study area including the main habitats and 95% Minimum Convex Polygon (MCP) of each fox drawn for both territorial and non‐territorial foxes (see Section 3 and Supporting Information). The MCP of seven of eight territorial foxes only includes human habitat at its periphery, and larger MCPs are on average more distant from human habitat. Trapping sites were all in the agricultural areas and villages within the home ranges of the territorial foxes.

Trapping of foxes was done in agriculture areas and villages in the southern Jordan Valley (the southern Arava Desert Latitude 29.7°–30.1° N, Longitude 34.9°–35.2° E, Figure 1). This is a hyper‐arid area with approximately 26.5 mm annual rainfall and mean monthly temperatures of 8°C–20°C (minimum–maximum) in mid‐winter (January) and 26°C–40°C in peak summer (August). Natural water sources in this area are extremely rare except for short periods after scarce rain events, and natural vegetation and animals are mostly limited to small, scattered, vegetation concentrations in dry riverbeds (Barocas et al. 2022). The natural diet of the Red Fox in these areas includes small mammals (e.g., Cape Hare *Lepus capensis* and smaller rodents), a variety of insects, reptiles, scorpions and small birds, alongside plants and fruits (Basuony et al. 2005). A few small villages, with a few hundred residents each, are scattered along the landscape, and a major high‐way crosses it north–south, alongside many smaller roads used mainly by farmers. Between these villages and along the valley are different agricultural areas, and a few small‐scale solar farms built in recent years. The agricultural areas are being irrigated throughout the year, attracting foxes and other species to the water and to the abundant prey and agriculture products. Among the attracted species are the Golden Jackal ( *Canis aureus* ) and the Wolf ( *Canis lupus* ), who compete with the fox over resources (Scheinin et al. 2006; Segev et al. 2021). These resources include seasonal vegetables and permanent crops, mainly palm plantations. The attraction of wild animals to these areas often leads to human–wildlife conflicts (Fehlmann et al. 2020; Nemtzov 2002). Animals damage human resources in search of water or food, while human habitat modifications change the local fauna and flora diversity (Fehlmann et al. 2020; Nemtzov 2002).

Using open‐source satellite imagery from Google Earth Pro, we drew polygons around three types of anthropogenic land uses in the area used by the foxes: agricultural areas, solar farms, and villages, including built areas regularly used by people such as petrol stations and shopping areas (Figure 1). We defined polygon borders along roads or fences that surround these areas, joining adjacent agricultural areas if they shared a road. This resulted in polygons ranging from 3 to almost 500 ha (Figure 1). Because solar farms were mostly inside agricultural areas, and considering the temporal resolution of our data, we were not able to separate solar farms and agricultural area use, and thus treated these together in all our analysis. While some fences exist for the abovementioned land uses, these fences do not deter foxes from entering or using these areas.

### 2.2 Fitting GPS Collars and Data Handling

During winter 2022–2023 we tagged 10 red foxes, six females and four males, with GPS‐GSM collars (Ornitela, 115 g, custom made), and tracked them for a period of between 2 months and 2 years (thus including all of the annual cycle). Foxes were caught using cage traps (79 × 28 × 33 cm, distributed by Green Place, Israel) to which they were lured with meat and chicken leftovers (English et al. 2026). The traps were checked 2–5 times per night. When a fox was trapped it was handled quickly by three people, without sedation: the eyes were covered to reduce stress, a collar with a GPS unit was installed on the neck and sex was checked visually. The collars were pre‐prepared with multiple size options, and the exact size was decided per individual fox. The full size of the collar was never used and unused collar material was cut off to reduce weight and drag. The entire procedure took 5–10 min between the time of arrival at the trap and the release of the fox at the trapping site.

GPS tags were programmed to acquire a location every hour between sunset and sunrise, and once during daytime, but only night‐time locations were used in the analyses. Tags were initially programmed to send data during the evening every 24 h and re‐programmed to send data every 3 days after we observed that the foxes behaved in an expected way and the tags sent data regularly. Jammed (spoofed) GPS data was manually removed by visually examining the data and identifying locations far from the tagging area, with no movement path leading to them (Jiguet et al. 2025). We then removed from our data 122 nights in which less than three locations were recorded. Finally, for the statistical analyses we only used data from eight of the trapped foxes, as two female foxes presented remarkably different behaviors, showing no consistent territory use (see Sections 3 and 4; Supporting Information). While this sample size constraints population‐level generalizations, it allows statistical inference that illuminates possible larger‐scale phenomena.

### 2.3 Statistical Analysis

To understand the rate of use of human habitats by foxes, we first annotated every GPS location with the habitat in which it was acquired—agriculture and solar, village or natural. We then calculated the rate of human habitats (agriculture, solar farms and village) visits by dividing the sum of nights in which foxes visited these habitats by the total number of nights with available data, per individual fox.

To examine the effects of environmental factors, we first obtained 10‐min temperature and relative humidity data from the Israel Meteorological Service website for Yotvata Weather Station, located at one of the villages visited by the foxes. For the same 10‐min time stamps, we added the proportion of moon illumination, using the R package “lunar.” We then acquired dusk and dawn times for every night at the study site using the R package “suncalc” and calculated nightly mean and maximum for each variable from dusk to dawn.

We tested the effects of the environmental variables on the probability that a fox will visit human habitats on a given night. To do so, we built a Generalized Linear Mixed Effects Model (GLMM) with a Binomial distribution and a logit link function. The response variable in this model was, for every night and every fox separately, whether a visit to human habitats occurred or not. Explanatory variables included mean moon illumination, total night length (calculated as the time between dusk and dawn), mean relative humidity, and maximum temperature (all continuous). Humidity and moon illumination vary along the night and thus we assumed that mean is the best representation of the factors that affect the foxes' movement. However, as temperature decreases along the night, we assumed that the maximum temperature is closer to the temperature experienced by the foxes when they initiate their movements, and thus provides a better measure than mean. To account for inter‐individual differences, we also included individual as a random intercept. Importantly, while temperature and relative humidity correlate, we did not find an effect of this correlation on our statistical models, with a variance inflation factor score < 1.6 for all variables.

To test for the relationship between the use of human habitat, energy expenditure and risk exposure, we used multiple proxies. First, we assumed that visiting human habitats allows increased energetic intake (Tucker et al. 2018). Conversely, we used movement measures to account for the expended energy, as longer movements entail higher energy expenditure (Covell et al. 1996; Shepard et al. 2013). Finally, we also assumed that moving over larger areas exposes the foxes to more risks (Laundre et al. 2010; Moleón et al. 2014; Oehler et al. 2025).

We then assessed the potential trade‐off between proxies for energy intake and expenditure by examining whether individual foxes' proportion of visits to human habitat was affected by the distance they were required to travel. We separated this analysis from the environmental variables model to isolate the effects of nightly environmental drivers from individual‐level spatial constraints. To that end, for each fox, we counted the number of nights with and without visits to human habitats and calculated the mean distance between the first location acquired each night and the closest human habitat for all nights, including nights with and without visits to human habitats. Then, we tested the effect of that averaged distance on whether a visit occurred or not using a GLMM with a Binomial distribution and a logit link function, including individual as a random intercept.

Finally, to investigate the effects of human habitats on energy expenditure and risk exposure, we examined the movement of foxes, testing two exploration metrics. We calculated, for every night of every fox, the direct distance between the two furthest locations (i.e., maximum distance) and the 95% minimum convex polygon (MCP). Then, to test the effects of human habitats on these metrics, we used two variables: whether a fox visited human habitat that night, and what was the minimum distance from the centroid of its nightly MCP to human habitat. Importantly, maximum distance and MCP are expected to be affected by the number of locations acquired per night, because few locations reduce the probability of calculating the real metrics. To overcome this, we first plotted these two metrics against the number of locations per night and visually located the maximum number of locations that affects them (Figure S1). Following this examination of data, we excluded nights with less than nine and eight locations from the analysis of maximum distance and 95% MCP, respectively.

We used two separate GLMMs with a gamma distribution and a log link function, one explaining maximum distance and the other explaining 95% MCP area. The explanatory variables in both models were whether the fox visited human habitat that night (categorical), the distance between the MCP centroid and the closest human habitat (continuous), and their interaction. The interaction term allowed us to evaluate whether behavioral shifts during visits to human habitat were moderated by the distance between a fox's core activity area and human habitat. The continuous variable was scaled and centered in both models, and both models also included individual as random intercept. The variance inflation factor score for all variables in these models was < 1.1.

Data handling, statistical analyses including model performance tests and visualizations were done using R software 4.4.1 (Brooks et al. 2017; Lüdecke et al. 2021; R Core Team 2021).

## 3 Results

All foxes were caught and tagged between December 7, 2022, and January 7, 2023 (Table S1). Individual fox data was received for 65–741 (min–max) nights. The 10 foxes presented two distinct behaviors: eight foxes being strictly territorial, remaining in the same area for the entire tracking period, and two presenting frequent exploratory movements (Figure S2). After removing the data from the two exploratory foxes and filtering out nights with less than three locations, the number of tracking nights ranged between 76 and 726 days (400 ± 222 mean ± SD), with a total of 574–6764 GPS locations (3280 ± 1986 mean ± SD) per fox, 3–13 locations per night (8.19 ± 2.37 mean ± SD) (Table S1). GPS collars transmissions ended for different reasons: three foxes died, one fox lost its collar probably due to human error when fitting the collar, four eventually stopped sending data probably due to drained battery, and two stopped with no clear reason (Table S1).

**FIGURE 2:**
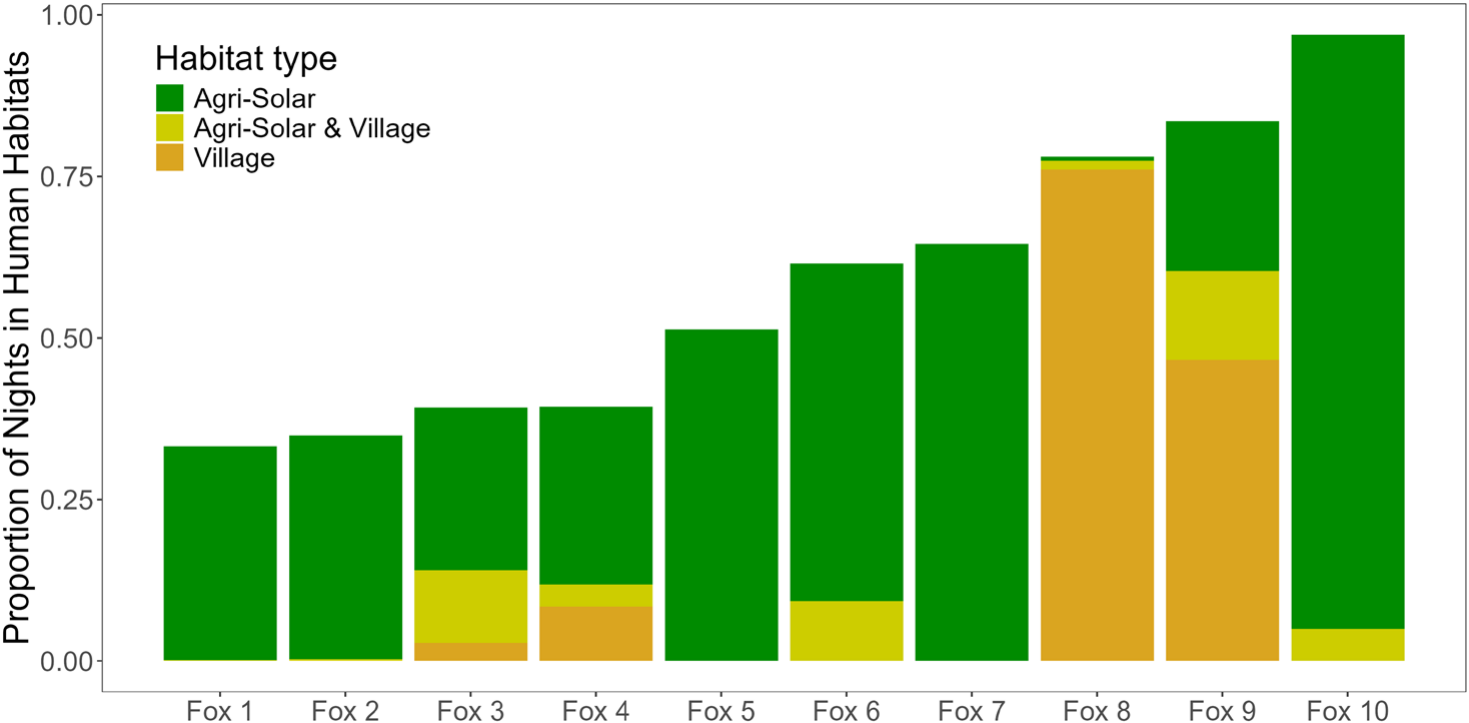
The use of human habitats by individual Red Fox is divided into the proportions of nights in which each fox visited agricultural areas and solar farms (Agri‐Solar, green), the proportion of nights in which it visited villages (orange) and the proportion of night it visited both habitat types (Agri‐Solar & Village, yellow). Foxes 4 and 6 were excluded from other analyses, as they did not present a territorial behavior (see Section 3).

Nine of the 10 foxes (all except for Fox 10) had most of their 95% MCP outside human habitats (Figure 1). The use of habitats differed between habitat types and individuals and the foxes usually visited more than a single habitat per night (e.g., visiting natural habitats during 99% of the nights does not mean visiting human habitats in just 1% of the nights). Seven of the 10 foxes were recorded in the natural habitat in all tracked nights; two foxes were recorded in natural habitats in > 99% of the nights (Fox 7 and Fox 8, 725 of 726 nights and 434 of 438 nights, respectively) and one fox in 92.8% of the nights (Fox 10, 451 of 486 nights). Overall, foxes visited human habitats in 33%–97% of the nights, with the preference for agriculture areas and solar farms or for villages differing among foxes (Figure 2).

**FIGURE 3:**
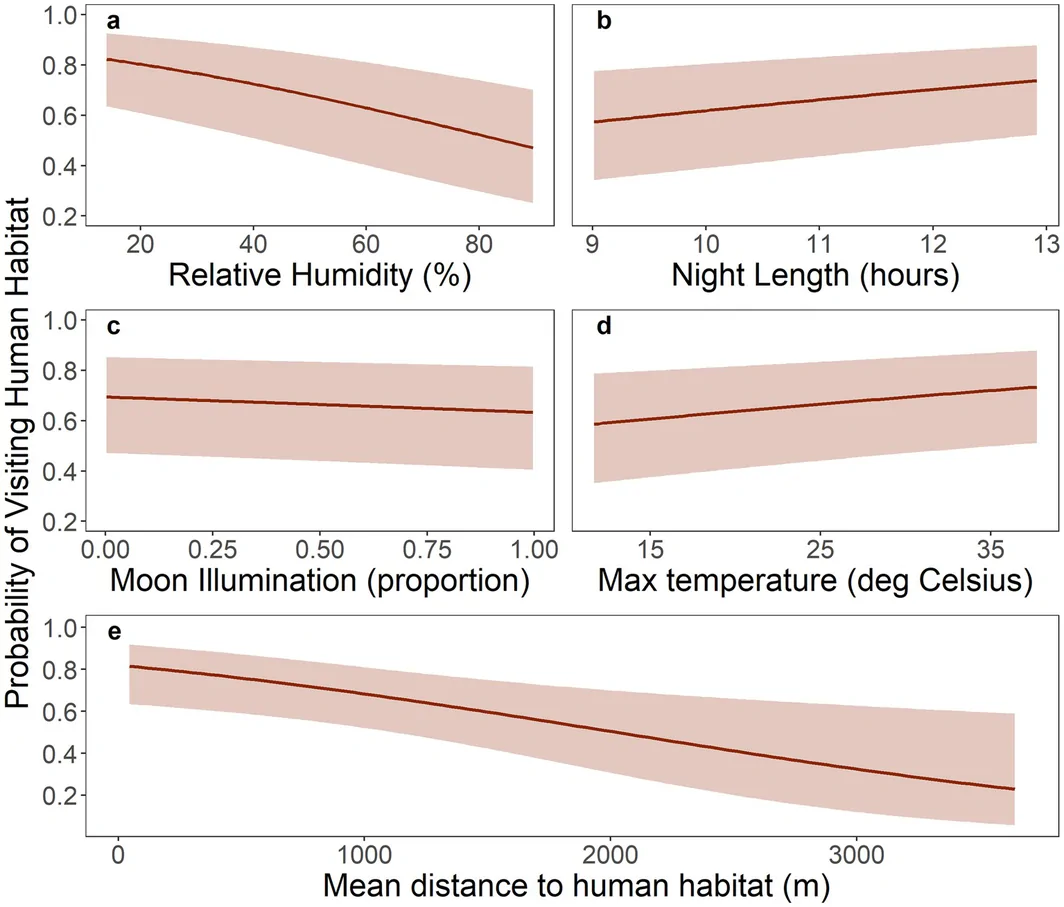
The effects of environmental variables on the probability that a Red Fox will visit human habitats in an extremely arid desert. (a) Mean relative humidity per night; (b) Time between dusk and dawn; (c) Mean moon illumination per night; (d) Maximum temperature per night; (e) Mean distance between the first recorded location for each fox each night and the closest human habitat. Lines present the effect of the variable listed on the *X* axis, predicted from a Generalized Linear Mixed Effects Model (Table 1), with all other variables held at their means. Colored areas around the lines are the 95% confidence intervals. The notable large confidence intervals likely result from the limited sample size (eight foxes) and the large inter‐individual differences in the proportion of visits to human habitats (Figure 2), with the random intercept variance being 1.75.

The probability of using human habitats at a given night was significantly affected by all environmental explanatory variables, but the effect sizes of all except relative humidity were small (Table 1, Figure 3a–d). Model results predict that the probability of using human habitats is reduced by 0.35 and 0.061 between the lowest and the highest mean nightly relative humidity and moon illumination measured during the study period, respectively (Figure 3a,c). Also, model results predict an increase of 0.15 and 0.16 in this probability between the lowest and the highest maximum nightly temperature and night length, respectively (Figure 3b,d). The mean distance between the first location acquired per night and the closest human habitat for every fox had a significant negative effect on the proportion of nights with visits to human habitats (Table 1, Figure 3e). Model results predict that this probability is reduced by 0.59 between the lowest and the highest mean distances.

**TABLE 1:** Generalized Linear Mixed Effects Model results for (1) the effects of environmental variables on the probability that a fox will visit human habitats; (2) the effect of the mean distance between the first location of every night and human habitat for each fox on the proportion of visits to human habitats.

	Estimate	SE	*z*	*p*
**Environmental effects**				
Intercept	−0.736	0.894	−0.823	0.41
Mean moon illumination	−0.273	0.123	−2.23	0.0257
Night length (dusk to dawn)	0.188	0.0503	3.74	< 0.001
Mean relative humidity	−0.0219	0.0042	−5.21	< 0.001
Maximum temperature	0.0255	0.0105	2.43	0.0152
**Individual distance effect**				
Intercept	1.52	0.484	3.14	0.00172
Mean distance to human habitat	−0.000752	0.000292	−2.57	0.0102

**FIGURE 4:**
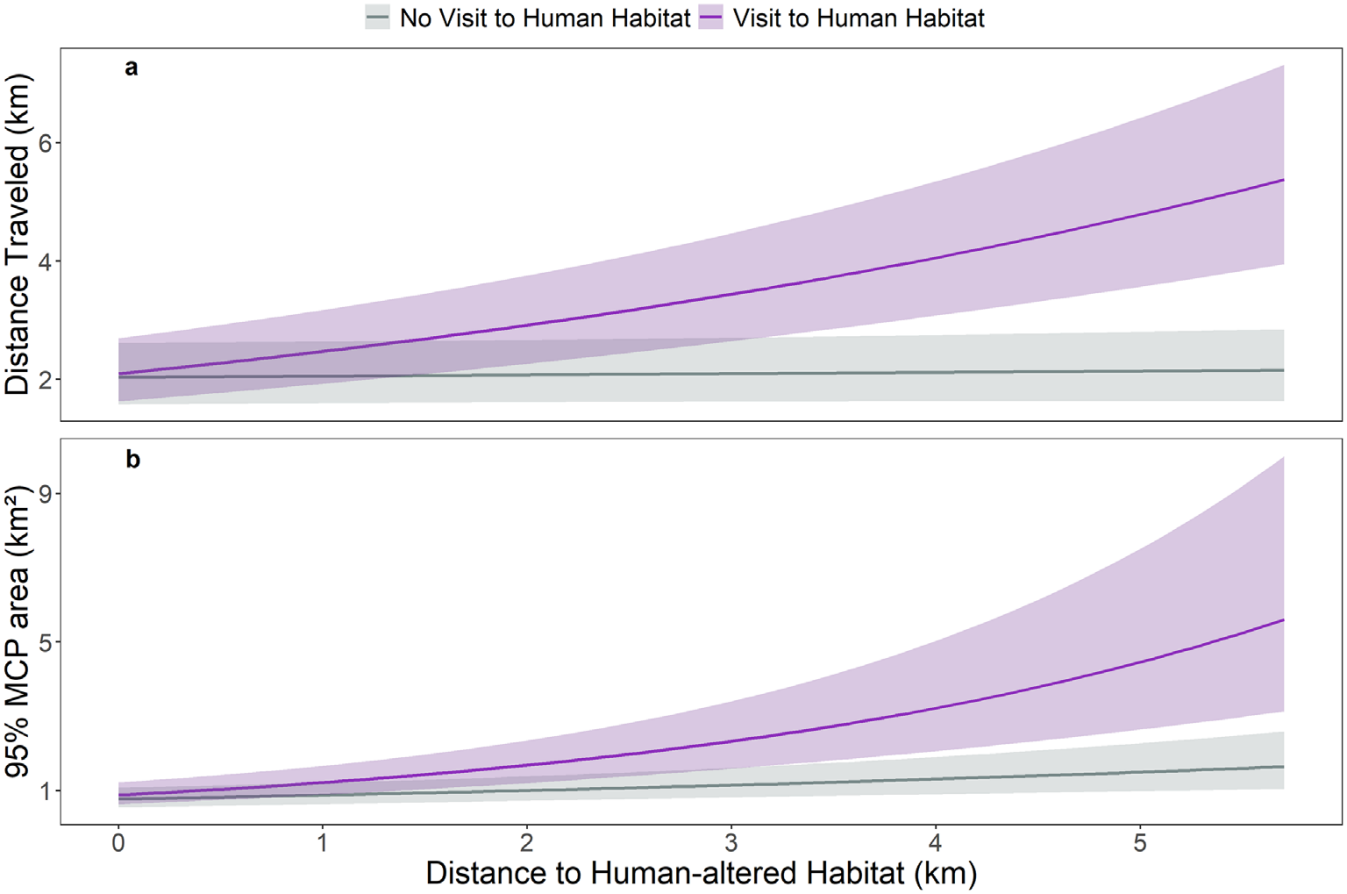
The effect of the interaction between whether a fox visited or did not visit human habitats and the distance of its Minimum Convex Polygon (MCP) centroid to the closest human habitat that night (a) on the maximum distance between two locations that night; (b) on the 95% MCP area. Lines present the effect predicted from a Generalized Linear Mixed Effects Model (Table 2). Colored areas around the lines are the 95% confidence intervals.

The number of nights used in the analyses of maximum daily movement and 95% MCP area were 2068 (258 ± 193 individual mean ± SD) and 1504 (188 ± 156 mean ± SD), respectively. Both movement metrics were significantly affected by whether a fox visited human habitat that night and by the interaction of this variable with the distance of the MCP centroid to the closest human habitat. The distance to human habitat also had a significant effect on MCP area but not on the maximum traveled distance (Table 2, Figure 4).

**TABLE 2:** Generalized Linear Mixed Effects Model results for the effects of the interaction between the distance of the Minimum Convex Polygon centroid to human habitat and whether a fox visited human habitats that night on (1) the maximum distance traveled and (2) the 95% MCP area.

	Estimate	SE	*z*	*p*
**Maximum distance**				
Intercept	7.63	0.128	59.8	< 0.001
Distance to human habitat	0.0144	0.0208	0.69	0.49
Visit to human habitat	0.213	0.0193	10.9	< 0.001
Distance × Visit	0.218	0.0197	11.1	< 0.001
**95% minimum convex polygon area**				
Intercept	13.7	0.164	84	< 0.001
Distance to human habitat	0.19	0.0553	3.44	< 0.001
Visit to human habitat	0.368	0.05	7.36	< 0.001
Distance × Visit	0.273	0.0541	5.04	< 0.001

*Note:* × represents an interaction term.

## 4 Discussion

The study of human–wildlife interactions has gained much attention in recent decades (König et al. 2020; Nyhus 2016). Using different tools, researchers have been able to shed light on variable aspects of these interactions and their impacts on wildlife. Here, we used tools from the field of movement ecology to study the use of human habitat by a widespread mammal, the Red Fox. By studying these interactions in a hyper‐arid environment, we were able to illuminate potential benefits and costs presented to a generalist species when taking advantage of abundant resources available in human habitats. Despite the relatively small sample size, we were able to show that, in our study area, foxes spend most of their time outside human habitats, only visiting these habitats on some nights. Furthermore, the probability of these visits depends on environmental conditions and the distance a fox needs to travel to get to these habitats. We highlight energy and risk costs related to these visits, revealing a likely trade‐off underlying the rate of use of human habitat by Red Fox in a hyper‐arid environment.

The hyper‐arid environment in which we studied foxes has substantial implications for the interaction of animals with human habitats. This environment imposes extreme challenges on animals, mainly due to the hot and dry climate and lack of water and food resources. Contrastingly, agriculture areas and human settlements provide year‐round water, abundant feeding options, and potential shelter. This contrast intensifies in high‐income settlements such as those in our study area (Chamberlain et al. 2020; Diamant et al. 2025). Our finding of increased rate of fox visits to human habitat with reduced relative humidity emphasizes the environmental stress in this hyper‐arid environment. However, we found much lower effects of temperature, moon illumination, and the length of the night and show that foxes do not visit human habitat every night. This suggests that foxes are not dependent on human habitats even in these extreme environments, to which they are probably adjusted through local physiological and behavioral adaptations (Geffen and Girard 2003; Willmer et al. 2009). A previous study found that the Red Fox population in our study area is genetically different from other geographically close populations that live in less extreme habitats, providing further evidence for suggested adaptations (Magory Cohen et al. 2013).

While species adaptation to arid environments can explain how foxes survive in these habitats without human‐related increases in available resources, it does not explain why they do not use these resources more. The Red Fox presents a strong commensal to human behavior across its range and throughout modern human history (Baumann et al. 2020), a trait known to positively affect population size and population density of many generalist animals, including the Red Fox (Clavel et al. 2011; Janko et al. 2012). This clear benefit is expected to further increase fox use of human habitat in our study area considering the difficulties presented by the natural environment. Thus, the observed low rates of visits by some of the foxes, as low as a visit every 3 days, suggest that the use of human habitats induces a substantial cost. This is further exemplified in the fact that only one fox had the center of its territory in human habitat, while the rest had human habitats at the edge of their territory. We suggest that the costs explaining this visit pattern are related to energy, risk, and territoriality (Gallagher et al. 2017).

Previous studies showed that Red Fox use of daily shelters and natal dens in human habitats and settlements is common, but limited by the availability of suitable micro‐habitats and competition (Harris 1981; Janko et al. 2012; Marks and Bloomfield 2006). While we did not find evidence for lack of shelter‐fitting micro‐habitat, the dense population of Golden Jackal ( *Canis aureus* ) in the agriculture areas of our study site (Segev et al. 2021) can act as deterring inter‐species competition. Furthermore, the strong territoriality and low overlap of the 95% MCPs between territorial foxes we found imply deterring intra‐species competition. Such deterring effects may force foxes to spend their days in varying distances away from human habitats, requiring longer movements that incur elevated energy and risk costs when using them (Shepard et al. 2013) and ultimately decreasing the use of these habitats. A direct energy cost consideration is supported by the strong reduction in visit rate with increased distance from human habitat. In a similar species, the Swift Fox ( *Vulpes velox* ), locomotion accounted for a major part of the daily energy expenditure, implying a major cost for increasing movement distances (Covell et al. 1996). Longer movements also possibly increase experienced risk through the rise in exposure to less familiar areas and the need to travel through dangerous areas, whether human (e.g., crossing roads, six of the territorial foxes had to cross a major highway to move between natural and human habitats) or natural (e.g., territories of conspecifics and other larger carnivores) (Laundre et al. 2010; Moleón et al. 2014; Oehler et al. 2025). A recent study presented different movement patterns of red foxes in different phases, stressing that the movement of foxes towards human habitats and in these habitats might be governed by different energy and risk considerations (Oehler et al. 2025). The rate of visits to human habitats by the two foxes that presented non‐territorial movements in our study was similar to the mean rate for all foxes despite their much longer movements compared to territorial foxes. This implies different trade‐offs regarding the use of human habitats for non‐territorial foxes, that should be further investigated with sufficient sample size.

Further evidence for energy and risk considerations is found in the comparison of movement during nights with and without visits to human habitat. We show that the distance traveled and the total occupied area per night (95% MCP) are similar among all foxes when they do not visit human habitats. Yet, when foxes do visit human habitats, these estimates of the range of movement increase by up to threefold with the distance from human habitat, coinciding with the threefold decrease in visit rate. This means that disregarding nights with visits to human habitat, the costs of movement are similar for all foxes. Thus, while proximity to human resources might reduce the mean traveled distance of foxes and other mammals (Main et al. 2020; Tucker et al. 2018), the underlying mechanism seems to be scale‐dependent. Our results suggest that, in our study system, two mechanisms might occur. In agreement with previous studies, foxes living in or close to human habitats might use the nearby resource‐rich habitat to reduce their movements compared to other foxes (Tucker et al. 2018). However, foxes leaving further away might be using the same habitat for landscape supplementation—allowing them to survive in their natural habitat by increasing their movement and taking advantage of human habitats (Dunning et al. 1992). Interestingly, landscape supplementation can also explain the increase in fox population density seen around human‐modified habitats. This supplementation allows animals to live in smaller and less resource‐rich home ranges, if they are able to travel the required distance to take advantage of the resource‐rich patches (Dunning et al. 1992). The energy and risk considerations presented here suggest that as distance increases, foxes will further reduce their use of human habitats, eventually avoiding them entirely. Whether such individuals move less or more than those studied here remains an open question (Main et al. 2020). The inconsistency in underlying mechanisms stresses the complexity of human–wildlife interactions and the necessity for habitat‐ and species‐specific studies to improve our understanding of these interactions (Cooke et al. 2019; Dharmarajan et al. 2021; Diamant et al. 2025; Sih et al. 2011).

Better understanding of animal behavior in response to human changes to the landscape is also important for the conservation of species and ecosystems (Berger‐Tal et al. 2011; Meaux et al. 2025), and for broader understanding of the ecological impacts of such behavior (Wilson et al. 2020). In addition to anthropogenic‐induced behavioral changes, our anecdotal observations of the cause of death by the three foxes for which we confirmed mortality agree with notions of high anthropogenic‐related risk in many other human habitats. One fox was shot in an agricultural area, another hit by a car on the way to human habitat, and the third diagnosed with severe sarcoptic mange, often assumed to be human‐related (Carricondo‐Sanchez et al. 2017). Thus, our results stress the elevated risk and energy expenditure related to the use of human habitats and should be considered when attempting to understand or manage human–wildlife interactions along human‐altered habitats.

## Author Contributions

**Ron Efrat:** conceptualization (lead), data curation (lead), formal analysis (lead), investigation (lead), methodology (lead), project administration (lead), validation (lead), visualization (lead), writing – original draft (lead), writing – review and editing (equal). **Nitzan Segev:** conceptualization (equal), investigation (equal), methodology (equal), project administration (equal), writing – review and editing (equal). **Oded Berger‐Tal:** conceptualization (equal), funding acquisition (lead), investigation (equal), methodology (equal), project administration (equal), resources (lead), supervision (lead), validation (equal), writing – review and editing (equal).

## Funding

This work was supported by the Israeli Ministry of Environmental Protection (164‐1‐2) and Alexander von Humboldt Foundation.

## Ethics Statement

The study was ethically supervised by Ben‐Gurion University of the Negev, under permit 2022/43165 from Israel Nature and Parks Authority.

## Conflicts of Interest

The authors declare no conflicts of interest.

## Supporting information


**Figure S1:** The effect of number of sampled locations per night on the (a) maximum nightly distance and (b) the 95% minimum convex polygon (MCP). Points are means for all nights and error bar are ±standard error.
**Figure S2:** Maximum daily distance histograms for individual foxes show two separate behaviors: a flat distribution with long movements for non‐territorial foxes and a closer‐to‐normal distribution for territorial foxes.
**Table S1:** Details of tagged foxes, tagging location and date, tracking duration and fate.

## Data Availability

Data and code are available in the Zenodo Data Repository at https://zenodo.org/records/19492862.
