# Supplementary material for: Use of Human Habitat in an Extreme Desert by the Generalist Red Fox: Risk and Energy Considerations
Source: Ecol Evol. 2026 Sep 16;16(9):e74392. doi: 10.1002/ece3.74392 (PMC13581412; doi:10.1002/ece3.74392)
Supplement: Supplementary file 1 [file ECE3-16-e74392-s001.docx]

**Supplementary Material**

**Figure S1**

The effect of number of sampled locations per night on the (a) maximum nightly distance and (b) the 95% minimum convex polygon (MCP). Points are means for all nights and error bar are ± standard error.

**Figure S2**

Maximum daily distance histograms for individual foxes show two separate behaviors: a flat distribution with long movements for non-territorial foxes and a closer-to-normal distribution for territorial foxes.

**Table S1**

Details of tagged foxes, tagging location and date, tracking duration and fate

| ID | Tag no. | First date | Last date | Tagging Longitude | Tagging Latitude | Sex | Tracking days | Location no. | Fate |
| --- | --- | --- | --- | --- | --- | --- | --- | --- | --- |
| 1 | 223933 | 21/12/2022 | 04/06/2024 | 35.072 | 29.937 | M | 531 | 5649 | Killed by an agriculture pest control hunter who misidentified it as a jackal |
| 2 | 223931 | 13/12/2022 | 06/11/2023 | 35.066 | 29.915 | F | 328 | 3658 | Found dead with scabies |
| 3 | 223930 | 13/12/2022 | 12/04/2023 | 35.071 | 29.938 | F | 120 | 1085 | Run over by a car |
| 4 | 223934 | 21/12/2022 | 04/10/2023 | 35.066 | 29.915 | F | 287 | 3104 | Tag fell or fox died. Tag probably underground, we couldn’t locate it |
| 5 | 223935 | 06/01/2023 | 01/04/2023 | 35.076 | 29.895 | F | 85 | 650 | Stopped sending data - unknown reason |
| 6 | 223936 | 06/01/2023 | 12/03/2023 | 35.061 | 29.884 | F | 65 | 779 | Tag fell - according to movement data and the state in which the tag was found |
| 7 | 223944 | 07/12/2022 | 17/12/2024 | 35.076 | 29.889 | F | 741 | 8069 | Disappeared - might be tag (battery) issues according to the long time it lasted |
| 8 | 223938 | 06/01/2023 | 01/05/2024 | 35.057 | 29.900 | M | 481 | 3622 | Disappeared - might be tag (battery) issues according to the long time it lasted |
| 9 | 223937 | 07/01/2023 | 21/07/2024 | 35.057 | 29.900 | M | 561 | 4374 | Disappeared - might be tag (battery) issues according to the long time it lasted |
| 10 | 223932 | 20/12/2022 | 14/05/2024 | 35.076 | 29.942 | M | 511 | 4739 | Disappeared - might be tag (battery) issues according to the long time it lasted |
